# 

**Published:** 1891-09

**Authors:** 


VOL. XII. SEPTEMBER, 1891. No. 9.

### THE

### International Dental Journal

A MONTHLY PERIODICAL

DEVOTED TO

### DENTAL AND ORAL SCIENCE.

**JAMES TRUMAN, D.D.S., Editor.**

Publication Office,
716 FILBERT STREET, PHILADELPHIA.

PUBLISHED BY THE

INTERNATIONAL DENTAL PUBLICATION COMPANY,

51 WEST THIRTY-SEVENTH STREET, NEW YORK CITY,
—AND—
716 FILBERT STREET, PHILADELPHIA.

Entered at the Post-Office at Philadelphia, and admitted for transmission through the mails at second-class rates.

$2.50 per Annum, in Advance. Single Copies, 25 Cents.

PRINTED BY J. B. LIPPINCOTT COMPANY, PHILADELPHIA.

